# Functional Gastrointestinal Disorders in Patients With Epilepsy: Reciprocal Influence and Impact on Seizure Occurrence

**DOI:** 10.3389/fneur.2021.705126

**Published:** 2021-08-06

**Authors:** Federica Avorio, Emanuele Cerulli Irelli, Alessandra Morano, Martina Fanella, Biagio Orlando, Mariarita Albini, Luca M. Basili, Gabriele Ruffolo, Jinane Fattouch, Mario Manfredi, Emilio Russo, Pasquale Striano, Marilia Carabotti, Anna T. Giallonardo, Carola Severi, Carlo Di Bonaventura

**Affiliations:** ^1^Epilepsy Unit, Department of Human Neurosciences, Sapienza University of Rome, Rome, Italy; ^2^Neurology Service, Therapeutic and Diagnostic Service Department, Istituto Mediterraneo per i Trapianti e Terapie ad alta specializzazione (IRCCS-ISMETT), Palermo, Italy; ^3^Neurophysiology Unit, Istituto di Ricovero e Cura a Carattere Scientifico (IRCCS) Neuromed, Pozzilli, Italy; ^4^Department of Physiology and Pharmacology, Istituto Pasteur-Fondazione Cenci Bolognetti, Sapienza University of Rome, Rome, Italy; ^5^Science of Health Department, School of Medicine, University Magna Graecia, Calabria, Italy; ^6^Institute for Research, Hospitalization, and Health Care (IRCCS) “G. Gaslini” Institute, Genoa, Italy; ^7^Medical-Surgical Department of Clinical Sciences and Translational Medicine, Sapienza University of Rome, Rome, Italy; ^8^Department of Translational and Precision Medicine, Sapienza University of Rome, Rome, Italy

**Keywords:** gut-brain axis, irritable bowel syndrome, dysbiosis, epilepsy, drug-resistance, functional gastrointestinal disorder, constipation

## Abstract

**Introduction:** The complex relationship between the microbiota-gut-brain axis (MGBA) and epilepsy has been increasingly investigated in preclinical studies. Conversely, evidence from clinical studies is still scarce. In recent years, the pivotal role of MGBA dysregulation in the pathophysiology of functional gastrointestinal disorders (FGID) has been recognized. With this background, we aimed to investigate the prevalence of FGID in patients with epilepsy (PWE) and the possible impact of bowel movement abnormalities on seizure recurrence.

**Methods:** A total of 120 PWE and 113 age-, sex-, and BMI-matched healthy subjects (HS) were consecutively enrolled. A questionnaire to evaluate the presence of FGID (according to Rome III diagnostic criteria) was administrated to all participants. In a subgroup of drug-resistant patients, we administered an *ad-hoc* questionnaire combining Bristol stool charts and seizure diaries to evaluate seizure trends and bowel movement changes.

**Results:** A higher prevalence of FGID in PWE (62.5%) than in HS (39.8%) was found (*p* < 0.001). The most frequently observed disorder was constipation, which was significantly higher in PWE than in HS (43.3 vs. 21.2%, *p* < 0.001), and was not associated with anti-seizure medication intake according to multivariable analysis. In drug-resistant patients, most seizures occurred during periods of altered bowel movements, especially constipation. A significant weak negative correlation between the number of days with seizures and the number of days with normal bowel movements was observed (*p* = 0.04). According to multivariable logistic regression analysis, FGID was significantly associated with temporal lobe epilepsy as compared with other lobar localization (*p* = 0.03).

**Conclusions:** Our clinical findings shed new light on the complex relationship between epilepsy and the MGBA, suggesting a bidirectional link between bowel movement abnormalities and seizure occurrence. However, larger studies are required to better address this important topic.

## Introduction

It is well-known that the digestive system and enteric neural structures have a close and reciprocal relationship with the central nervous system (1). Their interaction relies on two main pathways, namely the autonomic nervous system and the hypothalamic-pituitary axis, which crosstalk in a bidirectional way at different levels, while other players, including the gut microbiota and its products, contribute to the functioning of the entire system (2). All these components subserve a highly integrated system, the so-called microbiota-gut-brain axis (MGBA).

In recent years, research has focused on the impact of dysbiosis (3) and digestive disturbances on the development or progression of neuropsychiatric disorders, such as anxiety, depression (4, 5), autism and its spectrum (6, 7), neurodegenerative diseases (8–10), and epilepsy (11–13). Although the relationship between epilepsy and gastrointestinal disturbances has been investigated in preclinical studies (14, 15), approaching this crucial topic from a clinical perspective is a challenging task due to the number of interfering factors and complex variables typical of the real-world setting. Epileptic disorders can determine a multidimensional involvement of the gastrointestinal tract, including through ictal involvement of the brain structures subserving central control of autonomic functions (as suggested by the wide range of visceral symptoms), the possible interictal dysregulation of areas recurrently engaged by epileptic discharges, and the effects of anti-seizure medications (ASMs). This matter is even more hazy when considering the possible role of gut-dependent neuroinflammation and dysbiosis in the genesis of seizures and epilepsy (15). To shed light on such an intriguing issue, functional gastrointestinal disorders (FGID) could represent an ideal model by providing indirect information about gut-brain interactions according to the most recent pathophysiological conception (14). To investigate these interactions, we performed a clinical study exploring the prevalence and possible impact of FGID in a consecutive cohort of patients with epilepsy (PWE) as compared with healthy subjects (HS).

## Materials and Methods

From January 2015 to July 2019, we consecutively enrolled 120 adult PWE followed at the epilepsy outpatient clinic of Policlinico Umberto I of Rome. Age-, sex-, and body mass index (BMI)-matched HS were recruited during the same period. This study was conducted according to the Helsinki Declaration of Human Rights and informed consent was obtained from each participant.

The inclusion criteria were: (1) a confirmed diagnosis of epilepsy supported by clinical, neuroimaging, and electroencephalography (EEG) findings; (2) availability of complete and adequate medical documentation; (3) good compliance; (4) no organic gastrointestinal diseases or previous major gastrointestinal surgery; (5) no other medical or psychiatric comorbidities; and (6) absence of potentially interfering medication other than ASMs. HS were excluded if they had any history of medical or neuropsychiatric comorbidities and/or if they were taking any potentially interfering medication. We also excluded patients and HS who took any other dietary supplements or antibiotics within 4 weeks prior to the study.

For all enrolled patients, epilepsy characteristics were defined according to syndromic context, with specific regard to seizure type, etiology, and therapy response. In these cases, the lobe of seizure onset was defined based on clinical, interictal/ictal EEG, and anatomical (magnetic resonance imaging scan) findings. Drug resistance was defined according to International League Against Epilepsy (ILAE) criteria (16).

The main aims of our study were to define: (1) the prevalence of FGID in PWE as compared with HS; (2) the possible relationship between FGID and different epilepsy characteristics; and (3) the possible relationship between seizure occurrence and bowel movements. To this aim, the study was composed of two steps: (1) administration of a validated questionnaire for gastrointestinal symptoms following Rome III criteria (17) that was independently evaluated by two expert gastroenterologists (if present, symptoms were used to define the specific syndromic entities in the FGID spectrum) (17); and (2) evaluation of seizure trends paired to bowel movement patterns assessed by using a Bristol stool chart/seizure count (BSCSC)-integrated diary, planned *ad hoc* by the authors (see Supplemental Figure 1). Through this diary, we collected information about the relationship between seizure and bowel movements over 30 days in a subgroup of drug-resistant patients. Patients were asked to note the day of seizure occurrence (specifying the type) and the day of bowel movement, specifying the type of feces (Bristol score <3 was indicative of constipation, Bristol score of 3–5 was normal, and Bristol score >5 was indicative of diarrhea).

## Statistical Analysis

Data were tested for normal distribution using the Shapiro–Wilk test and data visualization methods. Data were presented as mean [standard deviation (SD)] or median [interquartile range (IQR)] as appropriate. We searched for a possible correlation between FGID and the different subcategories of PWE, such as gender, epilepsy syndrome (generalized, focal, or epileptic encephalopathies), disease duration (with a cut-off of 10 years), age of onset (more or <10 years), etiology (idiopathic/unknown or symptomatic), seizure rate (high or low frequency), therapy response (drug-sensitive or drug-resistant), drug burden (monotherapy or polytherapy), drug generation (first or second/third generation drugs), and seizure lobar localization at onset (frontal, temporal, parietal, or occipital). Comparison across relevant groups was performed using the Fisher exact test or the chi-square test. Group tests were two-sided, with a *p* < 0.05 considered statistically significant.

A multivariable logistic regression model was also elaborated to study the effect of different clinical and demographic variables on FGID. The presence of FGID was used as a dependent variable. Sex, epilepsy duration, drug resistance, structural etiology, ASM polytherapy at last observation, number of ASMs used during clinical history, frequency of seizures per month, and temporal lobe epilepsy (TLE) were used as covariates.

IBM SPSS Statistics version 25 for Windows (IBM Corp., Armonk, NY, USA) was used for data analysis.

## Results

### Clinical Characteristics of the PWE Population

The PWE population consisted of 120 patients (62 females). Median age was 41 years (IQR 29–54 years) and median BMI was 23.6 kg/m^2^ (IQR 21.6–27.7 kg/m^2^). Detailed demographic and clinical characteristics of the patient population are illustrated in Table 1. A total of 113 HS (59 females, median age 38 years, median BMI 24.2 kg/m^2^) were also enrolled for comparison. A detailed comparison of clinical-demographic data between patients and HS is represented in Supplementary Table 1.

**Table 1:** Demographic and clinical characteristics of PWE enrolled in the study (*N* = 120).

		***N* (%)**
Gender	Male	58 (48.3)
	Female	62 (51.7)
Age of onset	>10 years	83 (69.2)
	<10 years	37 (30.8)
Disease duration	<10 years	25 (20.8)
	>10 years	95 (79.2)
Epilepsy type/syndrome	Focal epilepsy	85 (70.8)
	Idiopathic generalized epilepsy	32 (26.7)
	Epileptic encephalopathy	3 (2.5)
Etiology of focal epilepsy	Structural or symptomatic	42 (35)
	Unknown/idiopathic	43 (35.8)
Lobar involvement in focal epilepsy	Temporal	39 (46)
	Extra-temporal	46 (54)
Therapy regimen	Polytherapy	81 (67.5)
	Monotherapy	37 (30.8)
	None	2 (1.7)
ASM generation	1^st^ generation	85 (70.8)
	2^nd^/3^rd^ generation	33 (27.5)
Drug-resistance	Total	71 (59.2)
	Focal	56 (46.7)
	Generalized	12 (10)
	Epileptic encephalopathy	3 (2.5)
Seizure rate in drug-resistant epilepsies	Annual/monthly	34 (28.3)
	Weekly/daily	37 (30.8)

*ASM, antiseizure medication; PWE, patients with epilepsy*.

### Prevalence of FGID

The prevalence of FGID was 62.5% (75/120) in PWE as compared with 39.8% (45/113) in HS (*p* < 0.001). Functional constipation was the most frequently observed disorder and was more common in the patient group, where it was present in 35.8% (43/120) of PWE, compared with only 15% (17/113) of HS (*p* < 0.001). The percentage of PWE with constipation [functional constipation plus irritable bowel syndrome (IBS) with constipation] was 43.3% (52/120), which was significantly higher than the percentage in HS (21.2%, 24/113) (*p* < 0.001). No statistically significant differences between patients and HS were found for any other FGID subtype. The prevalence of all FGID subtypes in patients and HS is shown in Table 2.

**Table 2:** Prevalence of FGID in PWE (*N* = 120) compared to HS (*N* = 113).

	**PWE**	**HS**	***p-value***
Total patients with FGID (at least one FGID)	75 (62.5)	45 (39.8)	<0.001
Dyspepsia subtype- post-prandial distress syndrome	13 (10.8)	13 (11.5)	n.s.
Dyspepsia subtype- epigastric pain syndrome	4 (3.3)	7 (6.2)	n.s.
Nausea and vomiting	0 (0.0)	3 (2.7)	n.s.
Belching	2 (1.7)	1 (0.9)	n.s.
Chest pain/heartburn	3 (2.5)	1 (0.9)	n.s.
Dysphagia	4 (3.3)	0 (0.0)	n.s.
Pharyngeal globe	3 (2.5)	0 (0.0)	n.s.
Regurgitation	1 (0.8)	0 (0.0)	n.s.
IBS	16 (13.3)	15 (13.3)	n.s.
IBS with diarrhea	3 (2.5)	4 (3.5)	n.s.
IBS with constipation and diarrhea	3 (2.5)	0 (0.0)	n.s.
Unclassified IBS	1 (0.8)	4 (3.5)	n.s.
IBS with constipation	9 (7.5)	7 (6.2)	n.s.
Functional constipation	43 (35.8)	17 (15)	<0.001
Total amount of patients with constipation	52 (43.3)	24 (21.2)	<0.001
Functional diarrhea	4 (3.3)	2 (1.8)	n.s.
Abdominal bloating	4 (3.3)	0 (0.0)	n.s.

*FGID, functional gastrointestinal disorder; HS, healthy subject; IBS, irritable bowel syndrome; PWE, patient with epilepsy*.

### Prevalence of FGID According to Seizure Localization, Epilepsy Type, Therapy Response, and Drug Burden

FGID was significantly more common in TLE than in other epilepsies. We found an FGID prevalence of 76.9% (30/39) in patients with TLE vs. 50% (23/46) in patients with extra-temporal lobe epilepsies (*p* = 0.02). See Table 3 for more detailed data about FGID prevalence stratified for all tested clinical variables.

**Table 3:** FGID according to demographic and clinical characteristics of the population.

	**Total**	**FGID+ (75)**	**FGID- (45)**	***p value***
	**120**	***N* (%)**	***N* (%)**	
**Sex**				
Male	58	33 (56.9)	25 (43.1)	n.s.
Female	62	42 (67.7)	20 (32.3)	
**Age of onset**				
>10 years	83	54 (65.1)	29 (34.9)	n.s.
<10 years	37	21 (56.8)	16 (43.2)	
**Disease duration**				
<10 years	25	18 (72.0)	7 (28.0)	n.s.
>10 years	95	57 (60.0)	38 (40.0)	
**Epilepsy syndrome**				
Focal	85	53 (62.4)	32 (37.6)	n.s.
Epileptic encephalopathy	3	2 (66.7)	1 (33.3)	
Idiopathic generalized	32	20 (62.5)	12 (37.5)	
**Etiology of focal epilepsies**				
Symptomatic	42	24 (57.1)	18 (42.9)	n.s.
Unknown/idiopathic	43	26 (60.5)	17 (39.5)	
**Response to therapy**				
Responsive	49	29 (61.2)	20 (38.8)	n.s.
Drug-resistant	71	46 (63.4)	25 (36.6)	
**Therapy regimen**				
Polytherapy	81	53 (65.4)	28 (34.6)	n.s.
Monotherapy	37	20 (54.1)	17 (45.9)	
None	2	2 (100)	0 (0.0)	
**ASM generation**				
First generation	85	48 (56.5)	37 (43.5)	n.s.
Second/third generation	33	25 (75.8)	8 (24.2)	
**Seizure rate in drug-resistant epilepsy**				
Annual/monthly	34	23 (67.6)	11 (32.4)	n.s.
Weekly/daily	37	23 (62.2)	14 (37.8)	
**Lobar involvement in focal epilepsy**				
Temporal	39	30 (76.9)	9 (23.1)	0.02
Extra-temporal	46	23 (50.0)	23 (50.0)	

*ASM, antiseizure medication; FGID, functional gastrointestinal disorder*.

According to multivariable logistic regression analysis, TLE was confirmed to be the only variable significantly associated with FGID (odds ratio = 2.62, 95% confidence interval = 1.06–6.51; *p* = 0.037). No effect was found for epilepsy duration, ASM, seizure status, or epilepsy etiology. The results of multivariable regression analysis are reported in Supplementary Table 2.

Regarding patients with constipation, no statistically significant differences were found in constipation prevalence between PWE receiving first generation vs. second or third generation ASMs or between PWE on mono vs. polytherapy.

### Relationship Between Seizure Occurrence and Bowel Movements

**Figure 1:**
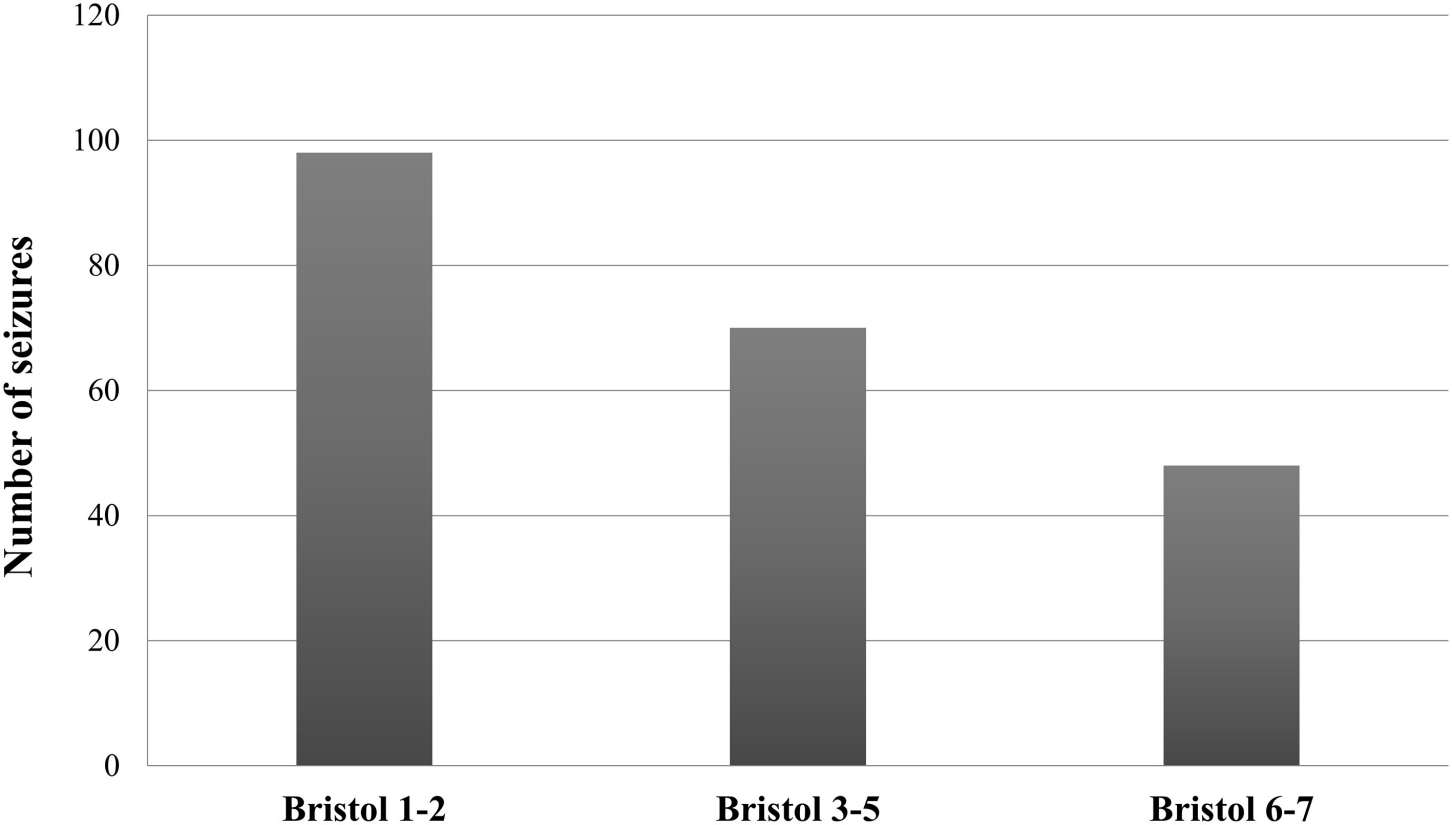
Seizure distribution according to Bristol categories in patients with drug-resistant epilepsy. The graph shows a more frequent occurrence of seizures during periods of altered bowel movements, especially constipation (Bristol score 1–2), as compared with periods of normal stools (Bristol score 3–5).

**Figure 2:**
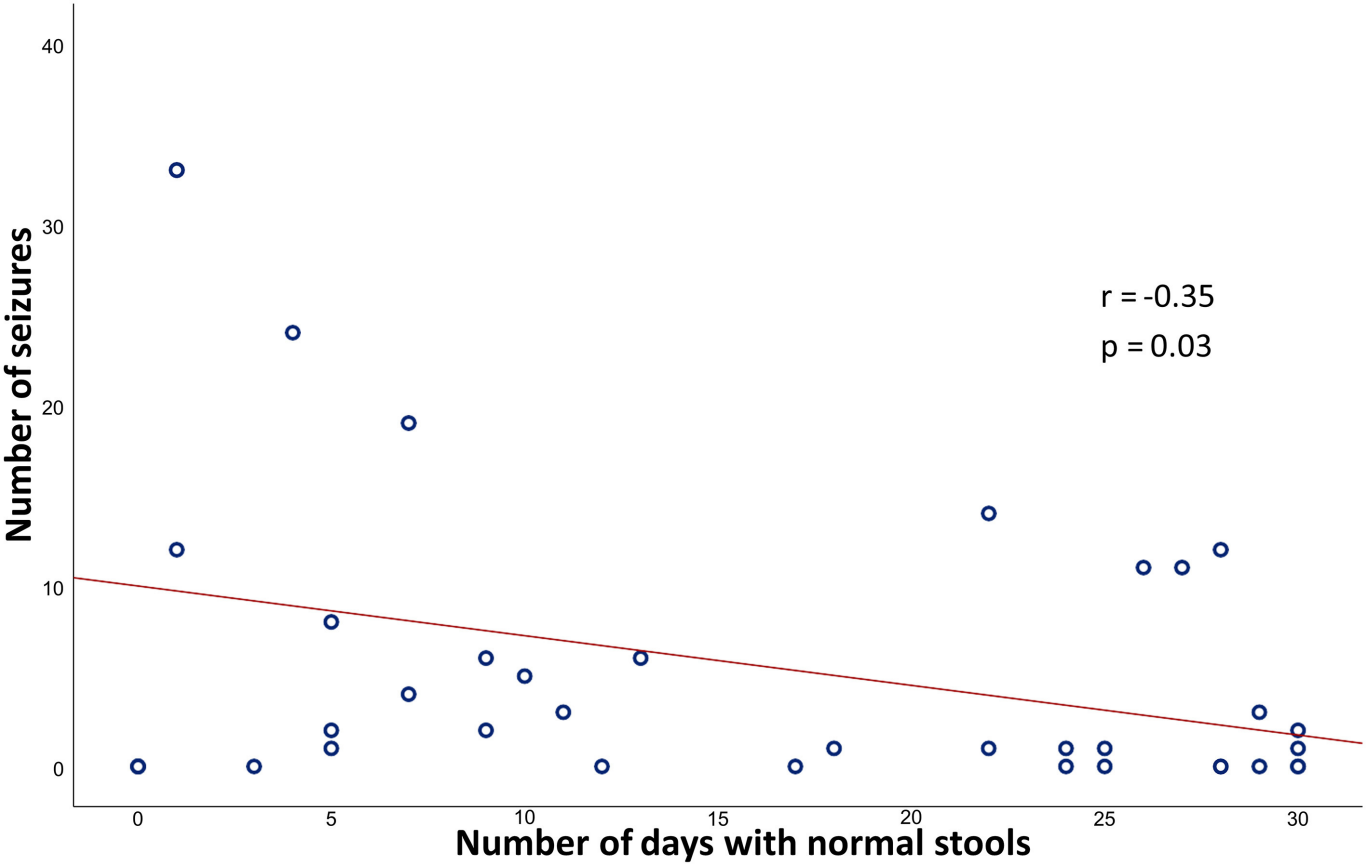
Pearson correlation coefficient (*r*) and *p*-value (*p*) between the number of seizures/month and the number of days with normal stools/month. The graph shows a significant negative correlation between the number of seizures/month and the number of days with normal stools/month.

A total of 38 patients with drug-resistant epilepsy correctly completed the BSCSC diary. Of the 216 total reported seizures, the majority (67.6%) occurred in a period of altered bowel movements. Specifically, 45.4% of seizures were reported during a period of constipation, while 22.2% were reported during a period of diarrhea (Figure 1). Moreover, we found a significant weak negative correlation between the number of seizures per month and the number of days with normal stools per month (*r* = −0.351, *p* = 0.03) (Figure 2).

## Discussion

This study represents a clinical contribution to better defining the relationship between the gut, brain, and epilepsy. Indeed, by analyzing different aspects of gastrointestinal function in PWE as compared with HS, the study revealed some interesting findings, including: (1) the higher prevalence of FGID in PWE as compared with HS, with constipation being the most common disorder; (2) the higher likelihood of seizure recurrence during periods of altered bowel movements (especially constipation) in drug-resistant patients; and (3) the higher frequency of FGID in subjects with TLE as compared with other lobar syndromes.

### FGID in Epilepsy

Despite the numerous factors possibly influencing our results, the clinical approach used in this study allowed us to confirm the relevance of FGID in PWE.

Constipation was the most common disorder in the examined cohort of patients. By analyzing a great number of variables, we were able to exclude a prominent effect of ASMs, whose role could be easily invoked. Indeed, we found no significant differences in FGID occurrence according to either drug burden (monotherapy vs. polytherapy) or ASM type (first vs. second/third generation ASMs), which were included in our multivariable analysis. However, given that only few patients were not taking any medication during the study period, we could not completely exclude the impact of ASMs on FGID by properly comparing subjects on and off ASMs, which clearly represents a limitation of our study. In analyzing the different FGIDs, it was not possible to confirm a higher prevalence of IBS in PWE as compared with HS as suggested by Camara-Lemarroy et al. (18). However, this discrepancy may be justified by the surprisingly low (3%) prevalence of IBS among control subjects in the aforementioned study. Conversely, our finding regarding IBS prevalence among control subjects was comparable with that reported in the general population by recent studies (19), strengthening the significance of our observation.

### Seizure Frequency Increases During Constipation

By reviewing the data collected through a targeted diary (namely the BSCSC diary), we observed that in drug-resistant PWE, seizures tended to occur or cluster mainly during periods of constipation. Likewise, a significant weak negative correlation was found between the number of days of normal stools and the number of seizures. This coincidence, often reported to physicians by epileptic patients themselves (and by subjects with migraine as well) (20, 21), seems to further support a close relationship between gut function and the brain. However, underlying influencing factors are yet to be elucidated, mainly due to the lack of rigorous/*ad hoc* studies conducted with a multidimensional approach, including careful metabolic assessment and microbiota extensive analysis. Consequently, establishing whether these bowel movement patterns influence seizures or vice versa is quite challenging. Studies performed on mice years ago showed a decreased seizure threshold after the administration of loperamide and/or clidinium (which decrease intestinal transit), suggesting the existence of a causal connection between constipation and seizure precipitation (22). Even if our study does not permit an exact cause-effect directionality between bowel movement abnormalities and seizure recurrence to be defined, the role of constipation appears to be an intriguing issue. In this case, a possible role of false neurotransmitters or other reabsorbed substances in facilitating cortical hyperexcitability and seizures could be hypothesized.

### Prevalence of FGID According to Seizure Localization, Epilepsy Type, Etiology, and Therapy Response

Another crucial aim of this work was to evaluate the possible influence of other variables (specifically epilepsy type, etiology, therapy response, and seizure localization) on gut function. While the analysis of patient subgroups according to epilepsy type (focal vs. generalized epilepsy), etiology (structural vs. unknown origin), and therapy response (drug-resistant vs. drug-sensitive) revealed no significant differences, interesting findings came from the comparison between patients with focal epilepsy and different seizure localization. Indeed, we documented a higher prevalence of FGID among patients with TLE, even after multivariable adjustment. These findings suggest that seizures and the underlying epileptogenic network could specifically contribute to determining gut function alterations in TLE patients. Indeed, the pivotal role of limbic structures in the anatomo-functional organization of the central autonomic network, a highly integrated system sub-serving the control of vegetative functions, is widely recognized (23). In this scenario, network disruption, either chronically- or acutely-induced by persisting seizures and underlying epileptogenic processes, could contribute to autonomic function dysregulation. On the other hand, it is well-known that TLE patients harbor a high rate of psychiatric comorbidities in comparison with other lobar epilepsies, with anxiety, depression, and alexithymia being the most frequent (24, 25). Considering the well-known role of psychiatric factors in the pathophysiology of FGID (26, 27), it is possible that the peculiar psychological characteristics of TLE patients might actually contribute to our findings. Unfortunately, we could not use specific questionnaires to assess such features in our cohort and adjust the statistical analysis.

The overall findings of the present study contribute to the historical debate regarding the reciprocal influence between the gut and brain in epilepsy (28, 29). Several factors, acting from bottom to top and vice versa, are thought to play a role in the genesis or maintenance of FGID in epilepsy. Indeed, microbiota-gut complex alterations can modify several neuronal functions by promoting or facilitating the inflammatory cascade, altered metabolism of neurotransmitters or their precursors, and dysregulation of gut permeability to bacterial fragments and products (30–32).

### Limitations of the Study

Although our results offered stimulating suggestions, the study had several limitations. First of all, the consecutive enrollment of patients determined a substantial heterogeneity of the population, which hampered the accurate stratification of patients according to homogeneous subcategories (such as specific syndromic entities or etiology). Another important limitation of this clinical observation was the lack of analysis of microbiota or other metabolic parameters. Since we started the study in 2015 before the publication of the more recent Rome IV criteria, the use of Rome III instead of Rome IV is another limitation.

In conclusion, our findings support further investigations of FGID in epilepsy in order to better define the role of the MGBA in causing or maintaining epileptic processes and to clarify the complex interactions between the gut-microbiota system and neurological disorders.

## Data Availability Statement

The raw data supporting the conclusions of this article will be made available by the authors, without undue reservation.

## Ethics Statement

The studies involving human participants were reviewed and approved by Sapienza University Ethics Committee. The patients/participants provided their written informed consent to participate in this study.

## Author Contributions

CD had full access to all the data in the study and takes responsibility for the integrity of the data and the accuracy of the data analysis. FA, EC, AG, and CD: concept and design. AM, MF, BO, MA, LB, GR, JF, MM, ER, PS, MC, and CS: acquisition, analysis, or interpretation of data. FA and EC: statistical analysis. AM, MF, BO, MA, LB, GR, JF, MM, ER, PS, MC, CS, CD, and AG: supervision. All authors contributed to manuscript revision, read, and approved the submitted version.

## Conflict of Interest

The authors declare that the research was conducted in the absence of any commercial or financial relationships that could be construed as a potential conflict of interest.

## Publisher's Note

All claims expressed in this article are solely those of the authors and do not necessarily represent those of their affiliated organizations, or those of the publisher, the editors and the reviewers. Any product that may be evaluated in this article, or claim that may be made by its manufacturer, is not guaranteed or endorsed by the publisher.
