# Supplementary material for: Functional Gastrointestinal Disorders in Patients With Epilepsy: Reciprocal Influence and Impact on Seizure Occurrence
Source: Front Neurol. 2021 Aug 6;12:705126. doi: 10.3389/fneur.2021.705126 (PMC8377227; doi:10.3389/fneur.2021.705126)
Supplement: Supplementary file 1 [file Table_1.DOCX]

| **Supplementary table 1. Main clinical and demographic characteristics of PWE (N=120) compared to HS (N=113)** | | | |
| --- | --- | --- | --- |
|  | **PWE** | **HS** | **p-value** |
| Age, median (IQR) | 41 (29-54) | 38 (28-54) | 0.5 |
| Sex, female (%) | 62 (50.4) | 59 (52.2) | 0.8 |
| BMI, kg/m^2^, median (IQR) | 23.6 (21.6-27.7) | 24.2 (21.4-26.4) | 0.8 |
| Active or previous smoking habit, n (%) | 33 (27.5) | 30 (26.5) | 0.9 |
| Alcohol consumption, n (%) | 50 (41.7) | 48 (42.5) | 0.9 |
| Abbreviations: BMI= body mass index; FGID = Functional gastrointestinal disorder; HS = Healthy subject; IBS = Irritable bowel syndrome; PWE = patient with epilepsy | | | |
