# Supplementary material for: Functional Gastrointestinal Disorders in Patients With Epilepsy: Reciprocal Influence and Impact on Seizure Occurrence
Source: Front Neurol. 2021 Aug 6;12:705126. doi: 10.3389/fneur.2021.705126 (PMC8377227; doi:10.3389/fneur.2021.705126)
Supplement: Supplementary file 2 [file Table_2.DOCX]

| **Supplementary table 2. Results of the multivariable logistic regression analysis to determine the variables associated with functional gastrointestinal disorders** | | | |
| --- | --- | --- | --- |
| Predictor | OR | 95% CI | p value |
| Female sex | 1.54 | 0.7-3.4 | 0.27 |
| Epilepsy duration | 0.99 | 0.96-1.03 | 0.74 |
| Seizures occurring at least monthly | 0.68 | 0.23-1.98 | 0.48 |
| Drug resistance | 1.07 | 0.39-2.91 | 0.89 |
| Structural etiology | 0.88 | 0.4-1.95 | 0.75 |
| Temporal lobe epilepsy | 2.62 | 1.06-6.51 | 0.037* |
| Number of AEDs tried during clinical history | 1.06 | 0.89-1.25 | 0.51 |
| AED polytherapy at last observation | 1.16 | 0.41-3.3 | 0.78 |
| Data are presented as Odds Ratio (OR) along with the 95% Confidence Interval (CI). The asterisks indicate statistically significant variables (p<0.05). Abbreviations: AED = Antiepileptic Drug | | | |
