# Supplementary figures and images for: Functional Gastrointestinal Disorders in Patients With Epilepsy: Reciprocal Influence and Impact on Seizure Occurrence
Source: Front Neurol. 2021 Aug 6;12:705126. doi: 10.3389/fneur.2021.705126 (PMC8377227; doi:10.3389/fneur.2021.705126)

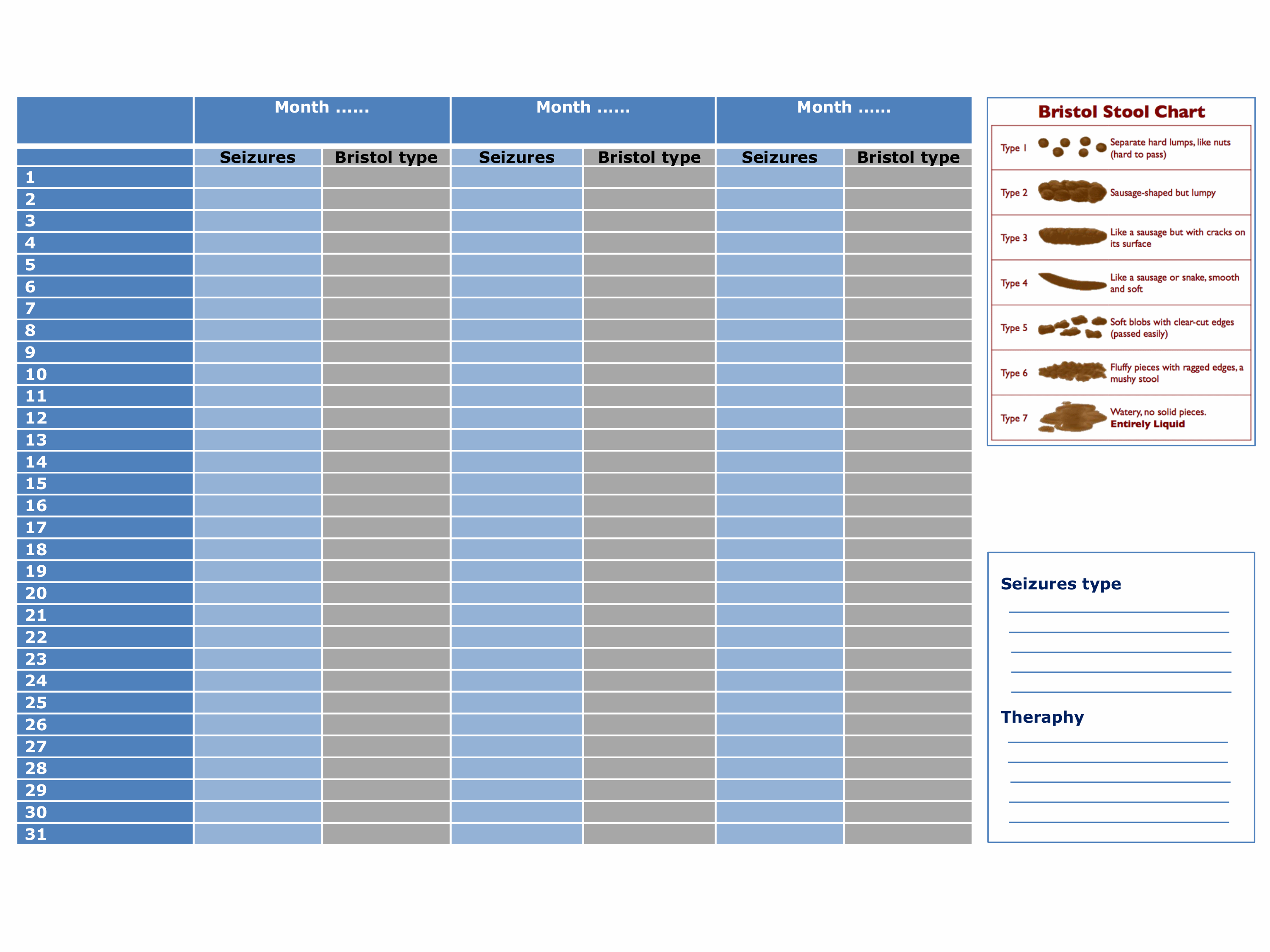

Supplement: Supplementary file 3 [file Image_1.TIF]
